# Examining the relationship between health-related quality of life and increasing numbers of diagnoses

**DOI:** 10.1007/s11136-015-1026-3

**Published:** 2015-06-12

**Authors:** Mathias Barra, Liv Ariane Augestad, David G. T. Whitehurst, Kim Rand-Hendriksen

**Affiliations:** Health Services Research Center, Akershus University Hospital, Postboks 1000, 1478 Lørenskog, Akershus Norway; Department of Health Management and Health Economics, Faculty of Medicine, University of Oslo, Oslo, Norway; Faculty of Health Sciences, Simon Fraser University, Burnaby, BC Canada; Centre for Clinical Epidemiology and Evaluation, Vancouver Coastal Health Research Institute, Vancouver, BC Canada

**Keywords:** EQ-5D, SF-6D, Comorbidity, Health-state utility value, Health-related quality of life

## Abstract

**Purpose:**

Little is known about estimating utilities for comorbid (or ‘joint’) health states. Several joint health state prediction models have been suggested (for example, additive, multiplicative, best-of-pair, worst-of-pair, etc.), but no general consensus has been reached. The purpose of the study is to explore the relationship between health-related quality of life (HRQoL) and increasing numbers of diagnoses.

**Methods:**

We analyzed a large dataset containing respondents’ ICD-9 diagnoses and preference-based HRQoL (EQ-5D and SF-6D). Data were stratified by the number of diagnoses, and mean HRQoL values were estimated. Several adjustments, accounting for the respondents’ age, sex, and the severity of the diagnoses, were carried out. Our analysis fitted additive and multiplicative models to the data and assessed model fit using multiple standard model selection methods.

**Results:**

A total of 39,817 respondents were included in the analyses. Average HRQoL values were represented well by both linear and multiplicative models. Although results across all analyses were similar, adjusting for severity of diagnoses, age, and sex strengthened the linear model’s performance measures relative to the multiplicative model. Adjusted *R*^2^ values were above 0.99 for all analyses (i.e., all adjusted analyses, for both HRQoL instruments), indicating a robust result.

**Conclusions:**

Additive and multiplicative models perform equally well within our analyses. A practical implication of our findings, based on the presumption that a linear model is simpler than an additive model, is that an additive model should be preferred unless there is compelling evidence to the contrary.

## Introduction

Economic evaluation of healthcare interventions is typically carried out using quality-adjusted life years (QALYs) as the outcome measure. The QALY combines length of life and health-related quality of life (HRQoL) in a single metric. As an addition to direct empirical comparison of QALY gain of available treatment options, modeling of cost utility is becoming increasingly common, since modeling based on existing data is more flexible and affordable than tailoring clinical tests to every scenario of potential interest. Such modeling rests on extensive use of preexisting recorded values representing the mean HRQoL loss associated with particular health conditions—so-called health-state utility values (HSUVs). This has created a demand for values for common ailments, which in turn has spurred on an effort to estimate catalogs of HSUVs associated with specific diagnoses [1, 2]. Priority setting in health care is becoming an increasingly important field for policy makers as the medical frontier is advancing ahead of budget constraints [3]. Access to sound estimates of health state utilities is important in order to ensure that resources are allocated in an efficient manner when evaluating treatments and interventions.

Modeling is complicated by the fact that patients frequently have more than one health problem, that is, are comorbid. Comorbidity is an ubiquitous and high-impact phenomenon [4], to the extent that three in four Americans above 65 years of age are diagnosed with two or more chronic diseases. In order to accurately estimate the QALY gain of alternative interventions in target populations, analysts need information about the HSUVs of health states characterized by being a combination of medical conditions.

Several efforts to construct catalogs of off-the-shelf HSUVs representing the mean HRQoL of various sub-populations have been undertaken [1, 2, 5]. These studies have in common that they are based on multivariate linear regression modeling on large datasets and thus can be said to incorporate quite accurate information while taking into account a number of factors which influence HRQoL. This is a suitable way for cost-utility modeling within a specific population where information (socio-demographics, diagnoses, etc.) is abundant. However, this method is not aimed at gaining knowledge about how comorbidities per se may interact with HRQoL. In particular, this paradigm assumes additive effects of having several diagnoses and therefore may be inadequate to inform on the relationship between comorbidity and HRQoL. A study by Sullivan et al. [6] looks at the impact of the number of chronic conditions on HRQoL, in a similar setting, concluding that the number of chronic conditions of an individual is a very important predictor of HRQoL.

A rather different approach to dealing with comorbidity is represented by attempts at identifying good mathematical models of comorbidity [4]. A mathematical model of comorbidity assumes that the HSUV of a compound health state can be estimated from the HSUVs of the component health states. Different models have been studied and compared, without any clear best fit [7, 8]. Research has mainly focused on combining single-state health state values into joint-state health state values [9–13], because large enough populations with any given combination of three distinct diagnoses are too small. Several joint health state predictors have been suggested (i.e., additive, multiplicative, best-of-pair, worst-of-pair, etc.), but no general consensus has been reached [7]. The various models (additive, multiplicative, minimum, etc.) lead to diverging predictions. An additive model implies that preferences should decline linearly with increased diagnoses; the multiplicative model implies diminishing marginal loss of HRQoL as a function of additional diagnoses. The best and worst-of pair models both imply a rapidly flattening HRQoL as diagnoses add up.

Investigating the mathematical relationship between single-state HSUVs and their corresponding joint-state HSUV is likely to be insufficient to uncover a general trend. Without any preconceptions about the preferred functional form, the purpose of this study is to explore the relationship between mean HRQoL and increasing numbers of diagnoses.

## Methods

### Data

We obtained data from the 2001 and 2003 Medical Expenditure Panel Survey (MEPS) [14]. These MEPS datasets contain detailed information on non-institutionalized US respondents’ health and socio-demographics, as well as self-reported HRQoL measured by two multi-attribute utility instruments, the EQ-5D and SF-6D (further details are provided below; the choice of years was based on the availability of contemporaneous EQ-5D and SF-6D data). The MEPS Web site also provides, in an auxiliary medical conditions file, a list of International Classification of Diseases, Ninth Revision, Clinical Modification (ICD9-CM) diagnose codes, which are linked to individuals by an identification variable. For privacy reasons, ICD9-CM diagnoses are provided as a truncated, 3-digit code in the MEPS file. For example, this means that an individual diagnosed with ‘hypertrophy of nasal turbinates’ (ICD9-CM code 478.0) and ‘polyp of vocal cord or larynx’ (ICD9-CM code 478.4) will be coded with two occurrences of the 3-digit ICD9-CM code 478.

### HRQoL instruments

The EQ-5D is one of the most frequently used instruments to assess HRQoL in health economic evaluation [15], requiring individuals to describe their health state across five dimensions: mobility, self-care, usual activities, pain and discomfort, and anxiety and depression. The 2001 and 2003 MEPS included the three-level version of the EQ-5D, in which each of the five dimensions has response options ‘no problems,’ ‘some problems’ or ‘extreme problems’. EQ-5D utility values were estimated using the preference-based algorithm published by Shaw et al. [16]. SF-6D scores were derived from the 12-Item Short-Form Health Survey (SF-12) [17]. The SF-6D is a multi-attribute utility instrument comprising items for the following six dimensions: physical functioning, role limitations (physical and emotional), bodily pain, vitality, social functioning, and mental health. Seven of the 12 items from the SF-12 are used to derive an SF-6D index score, and the six dimensions have between three and five levels of severity. SF-6D utility values were calculated according to the preference-based algorithm published by Brazier and Roberts [18].

### Inclusion criteria

We denote by *P*_*k*_ the collection of MEPS individuals who satisfy the following conditions: (1) at least 18 years of age, (2) have valid data for both HRQoL instruments, and (3) who have exactly *k* registered diagnoses in the MEPS medical condition file. Similarly, for an ICD9-diagnosis, *D,* the symbol *P*_*D*_ denotes the set of individuals who are at least 18 years of age, have valid HRQoL data, and are registered with diagnosis *D*.

### Statistical analysis

The primary aim of the study was to investigate how HRQoL is affected by additional medical diagnoses. To find support for generalizability beyond one specific HRQoL instrument, we analyzed both the EQ-5D and the SF-6D data from the MEPS dataset.

For each respondent in the MEPS 2001–2003 data, we computed an auxiliary variable named ‘Number of (registered ICD9) Diagnoses’ (NoD); a variable which simply counts the number of distinct ICD9 diagnoses assigned to the individual via the MEPS medical conditions file (*Note*: V-codes from the MEPS medical conditions files were omitted; this is further discussed in the Discussion section). Due to the 3-digit truncation of ICD9-codes, some responders were registered with more than one occurrence of the same ICD9-code. Such instances were counted with multiplicity, since they originate from different ICD9-codes in the underlying dataset. Next, the data were stratified according to the NoD variable into *P*_*k*_ subgroups. To ensure robust estimates of mean HRQoL for the NoD-defined strata, a pre-defined threshold of 1000 individuals, per strata, was required for inclusion in further analyses. For each strata satisfying this threshold, mean EQ-5D and SF-6D estimates were calculated. To assess the functional relationship between HRQoL and NoD, we next fitted three models to the aggregated data:

$${\text{Model A}}:\;{\text{ HRQoL}} = \alpha + \beta \cdot {\text{NoD}}$$

$${\text{Model B}}:\;{\text{HRQoL}} = \alpha + \beta \cdot {\text{NoD}} + \beta_{2} \cdot {\text{NoD}}^{2} \quad {\text{and}}$$

$${\text{Model C}}:\;{\text{HRQoL}} = \alpha \cdot \beta^{\text{NoD}}$$

Model A may support an additive—or linear—relationship. Model B may support a linear, an approximate multiplicative or an accelerating HRQoL loss relationship between NoD and HRQoL, depending on the signs, the magnitudes and the associated *p* values of the coefficients *β* and *β*_2_. Model C, which is equivalent to the model $\ln \left( {\text{HRQoL}} \right) = \alpha^{\prime } + \beta^{\prime } \cdot {\text{NoD}}$, reflects a multiplicative relationship between HRQoL and NoD. In all three models, the intercept (*α*) is interpretable as the estimated mean HRQoL of individuals with zero diagnoses. In Models A and B, a fixed decrement *β* is subtracted for each additional diagnosis; in Model B, an extra fixed adjustment of *β*_2_ · NoD is added to the estimate. For Model C, instead of a fixed decrement from *α*, the estimate is multiplied with a factor of *β* for each additional diagnosis; whence a good fit of this model may be taken as support of an underlying multiplicative relationship.

Because early inspection of plots of the values suggested the models would provide very similar fits, several model selection statistics were computed to explore our research question: regression coefficients, *p* values for the regression coefficients, the adjusted *R*^2^’s, the root-mean-squared error (RMSE), and the leave-1-out root-mean-squared residuals (L1O-RMSR) [19, 20], which is the analogous index of the model’s predictive value [21]. The residuals that enter the L1O-RMSR index are the distance between the predicted value and the observed value for HRQoL for *P*_*k*_, when leaving out the estimate of *P*_*k*_ when estimating the model (i.e., a standard leave-1-out cross-validation approach). As Model A is a nested specification of Model B, the two can be compared directly using standard analysis of variance methods, i.e., a nonsignificant regression coefficient for the quadratic term indicates over-specification. Because Models A and C are not nested models, there is no canonical best way of comparing them. As a further aid in interpreting the results, we also calculated the root-mean-squared distance (RMSD) between the fitted values of Model A and Model C. The RMSD is simply the Euclidean distance between the two models’ fitted values or equivalently the standard RMSE of Model A’s fitted values regarding Model C’s fitted values as the observed values. This last statistic is non-standard and therefore should be interpreted with caution. On the other hand, mathematically, the RMSD as defined here is simply the Euclidean distance between the fitted values of the two models, measured by the same metric as is used for the RMSE statistic. Therefore, it has one obvious interpretation: the relative differences in magnitude of the RMSDs between the two models, and the two models’ RMSEs, say something about the mutual distance between the fitted values relative to the fitted values to the observed ones. As for the regressions, all means for the RMSE’s and the RMSD’s were weighted by the strata’s relative sizes.

### Adjusting for age, sex, and severity

Correlations between demographic factors, such as age or gender, and HRQoL, or between the number of diagnoses and diagnosis severity, may introduce bias unless accounted for in the analysis. An ideal dataset would ensure that *P*_*k*+1_ is comprised of individuals from *P*_*k*_ after being affected by one more diagnosis. Within our dataset, this assumption does not hold because of inevitable differences in age, sex, severity, and numerous other factors. Accordingly, we performed a number of adjustments (age, sex, and severity) to compensate for these potential sources of bias. A detailed explanation of the adjustment methods is reported in Appendix. Briefly, for the severity adjustment, a new variable called ‘severity-weighted number of diagnoses’ (SWNoD) was computed for each respondent by summing severity weights rather than the (unadjusted) number of diagnoses. Note that the severity weights were calculated separately for EQ-5D and SF-6D, so that, for example, when investigating the relationship between SWNoD and EQ-5D, the severity weights used were computed with respect to the EQ-5D. Furthermore, for each of the HRQoL instruments, we computed severity weights using two sets of criteria. ‘Relaxed’ weights were calculated for all diagnoses for which we had at least one observation of an individual with no other diagnoses. The ‘strict’ weights required at least 10 sole-diagnosis individuals for a weight to be estimated.

In total, four analyses were carried out for each of the two HRQoL instruments (see Table 1 for an overview). All analyses were carried out in the statistical software R [22]; the models were fitted with the built-in linear regression modeling lm-function.

**Table 1:** List of analyses carried out to compare linear and multiplicative models

Analysis number	HRQoL instrument	Adjustment(s)
1	EQ-5D	None
2	EQ-5D	Age and sex
3	EQ-5D	Age, sex, and severity (‘relaxed’ definition)
4	EQ-5D	Age, sex, and severity (‘strict’ definition)
5	SF-6D	None
6	SF-6D	Age and sex
7	SF-6D	Age, sex, and severity (‘relaxed’ definition)
8	SF-6D	Age, sex, and severity (‘strict’ definition)

## Results

The pooled 2001–2003 material contains a total of 67,771 individuals. A total of 47,178 individuals were 18 years or older, out of which 39,817 (84.4 %) had valid data for both MAUIs (which were administered to 18+ year olds only). The age variable ranged over 18–85 (mean 45.36); 45.5 % were males. The NoD variable ranged over 0–45 (mean 3.28). A total of nine strata *P*_0_,…,*P*_8_ (consisting of patients characterized by having exactly 0,…,8 diagnoses) remained after omitting strata with fewer than a thousand respondents. Table 2 gives descriptive statistics for the strata *P*_0_–*P*_8_: unadjusted means for the strata’s mean HRQoL as measured by EQ-5D and SF-6D, mean age, percentage of males, strata size, and relative share (of the 39,817 with valid HRQoL information).

**Table 2:** Descriptive statistics for each stratum defined by the number of diagnoses

NoD	*n* (%)	Cumulative *n* (%)	Age	Male (%)	EQ-5D	SF-6D	NSWNoD^a^
0	7089 (17.8)	7089 (17.8)	36.9 (13.5)	56.9	0.948 (0.1)	0.862 (0.1)	0.000
1	7384 (18.6)	14,473 (36.4)	39.4 (14.6)	52.9	0.921 (0.1)	0.835 (0.1)	1.000
2	6194 (15.6)	20,667 (51.9)	42.4 (16.0)	50.2	0.893 (0.1)	0.811 (0.1)	2.029
3	4936 (12.4)	25,603 (64.3)	45.3 (16.9)	43.7	0.867 (0.2)	0.791 (0.1)	3.095
4	3624 (9.1)	29,227 (73.4)	48.9 (17.0)	39.6	0.838 (0.2)	0.769 (0.1)	4.182
5	2813 (7.1)	32,040 (80.5)	51.6 (17.4)	36.7	0.817 (0.2)	0.748 (0.1)	5.292
6	2093 (5.3)	34,133 (85.7)	53.4 (17.6)	35.0	0.785 (0.2)	0.723 (0.2)	6.435
7	1540 (3.9)	35,673 (89.6)	55.6 (17.3)	33.4	0.773 (0.2)	0.712 (0.1)	7.543
8	1212 (3.0)	36,885 (92.7)	58.2 (16.8)	31.5	0.742 (0.2)	0.686 (0.2)	8.713
Pearson’s correlation coefficients (*r*)			0.997	−0.979	−0.998	−0.998	1.000

Values are means (standard deviations) unless stated otherwise. The *r*-row reports Pearson’s correlation coefficients (*r*) between the number of diagnoses (NoD) and the mean values in the corresponding column. As a consequence of the a priori decision to exclude stratum with fewer than 1000 individuals, data for 7.4 % of the dataset were omitted from further analysis

*NoD* number of diagnoses, *NSWNoD* normalized severity-weighted number of diagnoses

^a^The derivation of the normalized severity weights is described in ‘Appendix’

The age distribution was skewed toward more elderly individuals in the strata representing more diagnoses, with a near-linear relationship between the strata’s mean over age and NoD. It is also the case that in general, the respondents with more diagnoses also have more severe diagnoses, as is evident by the perfect correlation (*r* = 1.000) between NoD and mean SWNoD (Table 2); indeed, the individuals with eight diagnoses have on average almost nine severity-adjusted diagnoses.

### Unadjusted analyses

For both HRQoL indices, the parsimonious linear models exhibited *R*^2^ > 0.995, indicating that a linear relationship between NoD and HRQoL explains the average values very well. Summaries of the regression models are presented in Table 3, together with results from the age-, sex-, and severity-adjusted variables.

**Table 3:** Key statistics for the regression models across the eight analyses described in Table 1

Analysis and model^a^	*α*	*β*	*β*_2_	*P*_2_	*Adj* *R* ^2^	RMSD^b^	RMSE	L1O-RMSR
1 A	0.9464	−0.0261	–	–	0.9972		0.0029	0.0051
1 B	0.9486	−0.0287	0.0004	0.0592	0.9983	0.0020	0.0021	0.0042
1 C	0.9488	0.9700	–	–	0.9983		0.0021	0.0037
2 A	0.9464	−0.0238	–	–	0.9972		0.0026	0.0046
2 B	0.9484	−0.0260	0.0003	0.0874	0.9981	0.0017	0.0020	0.0041
2 C	0.9484	0.9729	–	–	0.9981		0.0020	0.0037
3 A	0.9505	−0.0271	–	–	0.9974		0.0027	0.0049
3 B	0.9516	−0.0286	0.0002	0.3210	0.9975	0.0020	0.0025	0.0053
3 C	0.9527	0.9690	–	–	0.9974		0.0027	0.0043
4 A	0.9515	−0.0237	–	–	0.9962		0.0026	0.0060
4 B	0.9510	−0.0230	−0.0001	0.6261	0.9958	0.0014	0.0025	0.0089
4 C	0.9528	0.9734	–	–	0.9942		0.0032	0.0063
5 A	0.8582	−0.0220	–	–	0.9957		0.0030	0.0049
5 B	0.8609	−0.0251	0.0005	0.0198	0.9981	0.0016	0.0019	0.0041
5 C	0.8600	0.9723	–	–	0.9980		0.0020	0.0033
6 A	0.8583	−0.0212	–	–	0.9957		0.0029	0.0046
6 B	0.8608	−0.0239	0.0004	0.0391	0.9977	0.0015	0.0020	0.0043
6 C	0.8600	0.9735	–	–	0.9977		0.0021	0.0033
7 A	0.8613	−0.0224	–	–	0.9993		0.0012	0.0019
7 B	0.8621	−0.0234	0.0001	0.1134	0.9995	0.0016	0.0010	0.0017
7 C	0.8631	0.9719	–	–	0.9991		0.0013	0.0020
8 A	0.8612	−0.0201	–	–	0.9955		0.0023	0.0059
8 B	0.8621	−0.0215	0.0002	0.2913	0.9957	0.0011	0.0021	0.0058
8 C	0.8622	0.9752	–	–	0.9958		0.0021	0.0049

*Adj. R* ^2^ adjusted *R* ^2^, *RMSD* root-mean-squared difference, *RMSE* root-mean-squared error, *L1O-RMSR* leave-one-out root-mean-squared residual

^a^Model A is the linear/additive model, Model B the quadratic, and Model C the log-transformed/multiplicative model (for further details, see Methods section). Due to the model specifications, *β*_2_ coefficients are only relevant for Model B; *p*_1_ is the associated *p* value for the *β*_1_ coefficient. *β*_1_ coefficients for the three models, across all eight analyses, were significant at the 0.0001 level

^b^This statistic is the distance between the fitted values from Models A and C, analogous to the RMSE which is the distance between the fitted values and the observed values. The concept of distance is the standard (weighted) Euclidean distance between the sets of observed and/or fitted values

### Adjusted analyses

The regression model of EQ-5D as a function of age and sex within *P*_0_-stratum was significant for both independent variables (*p* < 0.000) and predicted age–sex reference values.

$$u_{\text{EQ}} \left( {a,s} \right) = 0.9697 - 0.0007 \cdot a + 0.0085 \cdot s$$

For SF-6D, only the sex variable was significant *s* (*p* < 0.000), and after leaving out the age variable (*p* > 0.05), the model predicted age–sex reference values as

$$u_{\text{SF}} \left( {a,s} \right) = 0.8487 + 0.0237 \cdot s$$

Of the 555 distinct ICD-9 diagnoses in the MEPS medical conditions file, there were 373 diagnoses for which a severity weight was obtainable from at least one individual (‘relaxed’ definition) and 124 diagnoses that at least 10 individuals had as their sole diagnosis (‘strict’ definition). After omission of individuals with non-weighable diagnoses, for the two different severity-adjustment criteria, the procedure retained 36,599 (91.92 %) for the relaxed inclusion and 25,858 (64.94 %) for the strict inclusion. The adjustments were non-trivial: The fraction of respondents who obtained a (rounded) SWNoD which differed from their originally computed NoD category ranged from 9.23 % (Analysis 8: ‘strict’ SF-6D-based SWNoD) to 44.97 % (Analysis 3: ‘relaxed’ EQ-5D SWNoD).

The computed model selection statistics (see Table 3) illustrate a good fit for all models, with very high adjusted *R*^2^ values throughout. The RMSE column shows that all three models give good fitted versus observed values. Furthermore, the quadratic Model B, with its additional parameter, tends to outperform the two other models with respect to this metric. The L1O-RMSR gives a different picture: Here the Model B under-performs, suggesting over-specification. The Model C performs slightly better than the Model A according to the L1O-RMSR metric; however, this gap is closed after adjusting for age, sex, and severity. In the L1O-RMSR metric, all models improve their fit as adjustments are made, except for analyses 4 and 8 which correspond to the strict inclusion. The RMSD column reports the distance between the predictions of the Models A and C. This column shows that the difference between the two models’ predictions is smaller than the difference between the two models’ respective predictions and the observed values.

**Fig. 1:**
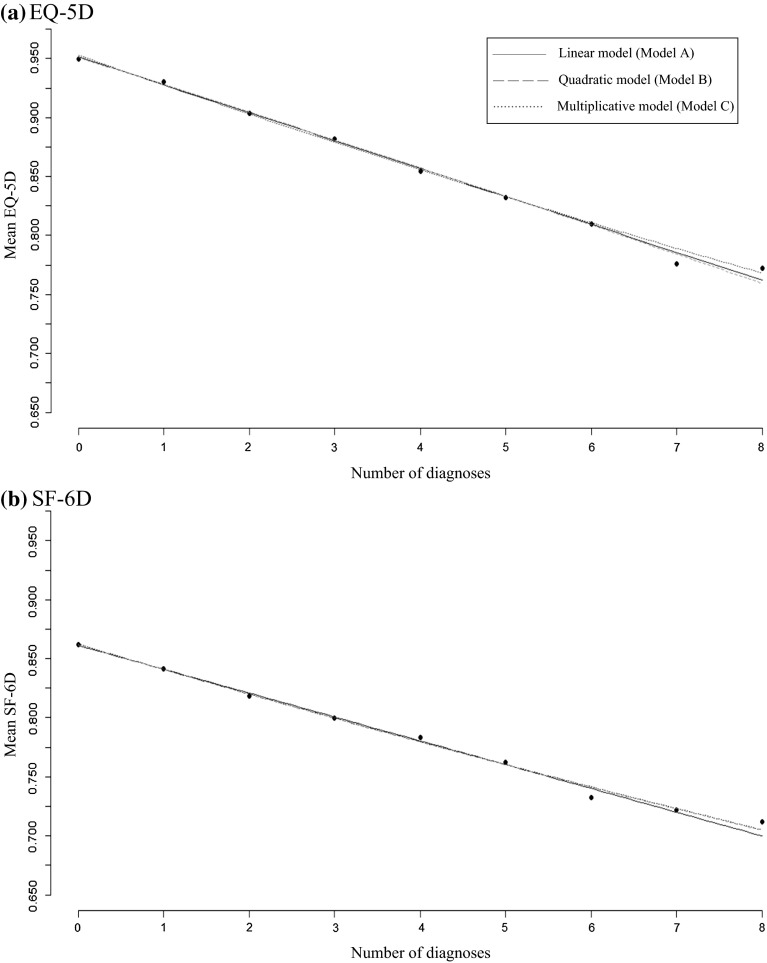
Illustration of model fit for Models A, B, and C for the fully adjusted analyses (age, sex, and severity) for the EQ-5D (**a**) and SF-6D (**b**). With reference to Table 3, **a** corresponds to analyses 4-A, 4-B, and 4-C; **b** corresponds to analyses 8-A, 8-B, and 8-C

For better visualization of the results presented in Table 3, Fig. 1a, b provides a graphical image of two of the models (Models 4A–C and 8A–C). We see that in both cases, the three Models A–C provide similar fits and that the immediate impression is that the parsimonious linear model describes the trend well.

## Discussion

The most striking property of the result reported in Table 3 is the similarity between the three models. Models A, B, and C display very similar fit indices, and the RMSE values suggest that all three models estimate the data well. Before adjustments for age, sex, and severity, Models B and C slightly improve the fit compared to the linear Model A. As expected, with its one extra degree of freedom, the quadratic Model B tends to beat the two other with a few thousands of a unit; however, looking to the L1O-RMSR column, it appears over-specified. After adjustments are made, Model A outperforms or matches Model C.

Examining the RMSE and L1O-RMSR for Models A and C does not identify either as being superior. If we assume that the adjusted analyses are the most appropriate, the improved fit of Model A suggests a possible underlying true additive relationship. The results also suggest that Models A and C are more similar to each other than to the underlying data, as reflected by the RMSD values being smaller than the two models’ RMSE statistics.

On average, little is gained from adding a quadratic term to a linear model for predicting HRQoL loss associated with extra diagnoses. This suggests that the general trend, on average, is adequately captured by a linear model. That this in conflict with many studies from the joint-state literature may be due to the fact that an additive model, working directly with the HRQoL losses associated with a single-state condition, does not account properly for the HRQoL loss present also among those with no diagnoses.

### Strengths and limitations

While several studies investigating the impact of having two simultaneously existing diagnoses (the joint-state literature) have been carried out [9–13], the more general and underlying question of how diagnoses impact HRQoL has not been previously addressed. The study undertaken by Sullivan et al. does to some extent overlap with this study because they both incorporate respondents with multiple diagnoses. However, whereas Sullivan’s model is designed to predict individual HRQoL, given rich information about the individuals’ age, sex, diagnoses, and other covariates, our model is solely focusing on the independent impact of diagnoses on HRQoL. Put simply, Sullivan focuses on the HRQoL of individuals, with a rich model, while we use a sparse model to focus on the functional relationship between the number of diagnoses and HRQoL.

Previous studies [9, 11–13] have used the clinical classification categories (CCCs) as a crude measure of disease. The CCCs also include V-codes; ‘supplementary Classification of Factors Influencing Health Status and Contact with Health Services (V01.0–V91.99) is provided to deal with occasions when circumstances other than a disease or injury (Codes 001–999) are recorded as a diagnosis or problem’ [23]. As such, V-codes carry with it information about other factors than morbidity qua morbidity. Using the truncated CCC information—or defining NoD-stratum—without omitting the V-codes thus may lead to groups with possibly biased HSUV values. The working directly with the ICD-9 diagnoses in this study permits omitting V-codes and is a strength of our analyses.

The interpretation of our results depends on patients with *n* + 1 diagnoses being comparable to patients with *n* diagnoses with the exception of the additional health problem. The major concern is that patients with more diagnoses may be afflicted with problems of different severity from the ones with patients with fewer diagnoses. However, the extent to which this is a problem for our analyses directly transfers to all attempts at determining the functional form for addition of health problems. Adjusting for severity goes some way toward ensuring such comparability. Still, the validity of our findings regarding the relationship between number of diagnoses and HSUVs depends on the generalizability of the MEPS data with regard to that relationship. Our analyses are made under the assumption that sampling error and missingness are random with respect to the functional relationship under scrutiny.

Since we did not gather the data ourselves, we have limited control of the quality of the data. However, it is unlikely that there should be any systematic biases in the collection process that would affect HRQoL values as a function of NoD. The data were collected in an outpatient setting, meaning that we cannot necessarily generalize to, e.g., a hospitalized population.

Even though our analyses are carried out on mean values computed over populations with 1000+ members, only nine strata were included. This means that the linear relationship observed may not describe the actual trend for patients with nine or more diagnoses. We do not suggest that the regression models are useful in themselves, only that they help investigate the underlying relationship between morbidity, as measured by diagnoses, and HRQoL.

The observed range of mean HRQoL values in our sample (0.948–0.742 for EQ-5D and 0.862–0.686 for SF-6D) may limit our ability to distinguish between the predictions from the additive and the multiplicative approaches. The problem could be ameliorated by looking specifically at severe diagnoses, but this would come at the cost of substantially reducing the number of available observations. As it is, the observed range of HRQoL values is based on more than 93 % of the population sample, suggesting that we are covering most of the relevant range of disease in the population.

## Conclusions

The three model specifications explored in this analysis—the linear (A), the linear with a quadratic term (B), and the multiplicative (C) (see the "Methods" section)—were virtually identical, indicating that a linear model adequately represents the trend on average. Occam’s razor suggests that the simplest model should be preferred. On this basis, we recommend discontinuing the search for a general multiplicative model. The study does not support the general notion of declining marginal disutility of health.

The observation that the average over thousands of patients with hundreds of different diagnoses match a linear function through number of diseases does not indicate that there exists a general linear model that can predict the mean HRQoL for a given combination of diagnoses from the HRQoL of the constituent diagnoses; the averages in question collapse a wide distribution of diagnoses that mask each other, exacerbate each other, or behave erratically in combination. The use of any general model, including the additive, is likely to lead to predictions that deviate substantially from reality in most cases even if the deviation is unbiased across studies. We recommend using empirical estimates of the HRQoL for patient groups with combination health states where this is possible. When such estimates are unattainable, any non-empirical estimates should be made based on expertise that allows predictions of the manner in which the constituent health problems should be expected to interact.

## Figures and Tables

**Fig. 2:**
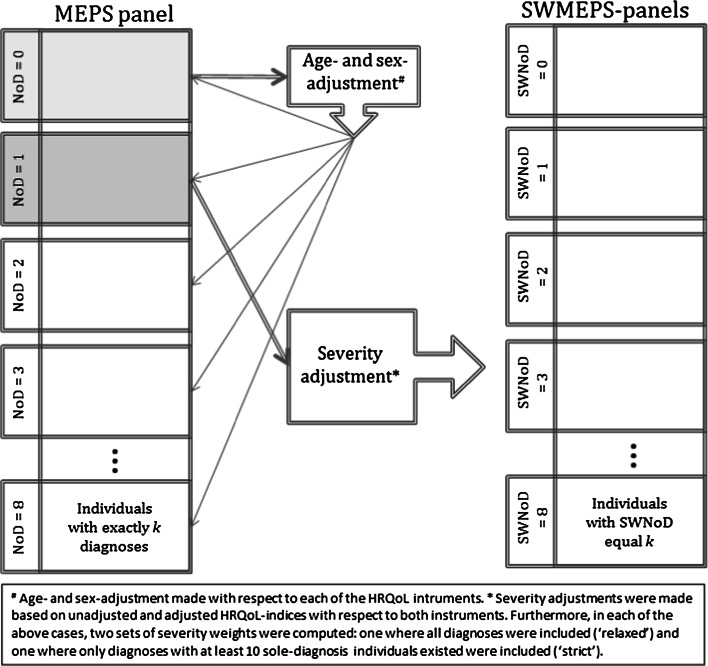
Schematic of adjustments for age, sex, and severity of diagnoses. Arrows show dependencies. Age and sex adjustment is made on the basis of the *P*_0_ stratum, prior to adjusting for severity on the basis of the *P*_1_ stratum. Following the severity adjustment of diagnoses, we have a new stratification of the dataset (indicated on the right-hand side)

## Appendix: Details of the age, sex, and severity adjustments

### Age and sex adjustment

Age- and sex-adjusted HRQoL values were computed, for the EQ-5D and SF-6D, in the following way. In step 1, a linear regression model was fitted to the *P*_0_ stratum (i.e., those individuals with zero diagnoses):

$${u_i} = {\beta_1} + {\beta_2} \cdot {a_i} + {\beta_3} \cdot {s_i} + {\epsilon_i}$$

where *u*_*i*_ is individual *i*’s HRQoL (either EQ-5D or SF-6D), *a*_*i*_ is age (in years), and *s*_*i*_ is a sex-dummy. In step 2, the estimated age- and sex-specific reference value (for each pair *a* and *s*)

$$u_{a,s} = \beta_{1} + \beta_{2} \cdot a + \beta_{3} \cdot s$$

yielded age- and sex-specific deviancy from the mean HRQoL for the *P*_0_-stratum (*u*_0_) by:

$$u_{0} - u_{a,s}$$

This enabled us to define an age- and sex-adjusted (ASA) HRQoL values (*u*′) for each individual:

$$u^{\prime}_{i} = u_{i} + \left( {u_{0} - u_{{a_{i} ,s_{i} }} } \right)$$

This approach does not attempt to minimize residuals on HRQoL given age and sex for the whole MEPS panel. Rather, it assumes that there is an independent effect of age and sex on HRQoL, which does not interact with diagnoses. Once the effect has been estimated, we adjust for it by taking away from (or giving back to) all individuals the HRQoL gained (or lost) as a result of their age and sex. The reason for restricting the regression model that estimates the age- and sex-specific HRQoL decrement to *P*_0_ is that this stratum is where the specific age and sex impact on HRQoL is disentangled from that of the diagnoses. It may help to understand the adjustment by considering that when individual *i* belongs to an age–sex class with higher predicted HRQoL than the average zero-diagnosis individual ($u_{0} < u_{{a_{i} ,s_{i} }}$), then *i*’s HRQoL will be decreased by $u_{0} - u_{{a_{i} ,s_{i} }} > 0$. Inverting the inequalities shows that individuals with lower-than-average reference value for their HRQoL will have increased HRQoL as with this adjustment.

### Severity adjustment

Severity weights were computed for the various diagnoses contained within the MEPS dataset by performing subgroup analyses on individuals with exactly *D* as their diagnosis, i.e., respondents in the *P*_1_ stratum. However, age and sex differences between diagnosis-defined subpopulations could also interfere. If some diagnosis subgroups were predominantly made up of young males and others of older females, the presence of age and/or sex gradients for HRQoL could bias the computed severity weights, so the procedure described below was performed with respect to age- and sex-adjusted HRQoL values.

In step 1, we excluded all individuals who had a diagnosis that did not occur as the sole diagnosis of at least one respondent in the MEPS panel. In step 2, based on respondents with exactly one diagnosis, we estimated the mean HRQoL loss, *d*_*D*_ (for both the EQ-5D and SF-6D), associated with each diagnosis. In step 3, a severity weight was assigned to each diagnosis:

$$s_{D} = d_{D} /d$$

where *d* is the average over all the *d*_*D*_’s computed in step 2. This operation results in a normalized severity weight such that *s*_*D*_ > 1 means that the diagnosis has a greater-than-average impact on HRQoL, and *s*_*D*_ < 1 has a less-than-average impact.

Step 1 ensures that all remaining individuals may have a severity weight assigned to all of their diagnoses. Note also that steps 2 and 3 result in one distinct severity weights set for each HRQoL instrument (in our case, EQ-5D and SF-6D). Step 1 necessitates discarding individuals who have diagnoses that are non-weighable (because no one has only that diagnosis; hence its independent impact on HRQoL cannot be estimated). The subsequent re-stratification (described below in step 4) resulted in somewhat smaller strata. Fourteen of the 18 strata retained more than one thousand individuals; the exceptions were stratum *P*_8_ (*n* = 763) under the relaxed inclusion, and strata *P*_6_ (*n* = 721), *P*_7_ (*n* = 431) and *P*_8_ (*n* = 272) under the strict inclusion.

In step 4, a new variable ‘severity-weighted number of diagnoses’ (SWNoD) was computed for each respondent by summing severity weights rather than the (unadjusted) number of diagnoses. For example, a respondent with three diagnoses *D*_1_, *D*_2,_ and *D*_3_ with severity weights 0.9, 0.95, and 1.70 would obtain a raw SWNoD of 3.55, reflecting the increased severity. SWNoD values are subsequently rounded to the nearest integer to permit re-stratification of the data.

Appendix Fig. 2 provides a schematic representation of the age, sex, and severity adjustment methods.

For further motivation for why the severity adjustment is important, particularly when dealing with our research question, consider the following example:

**Table:**

Example: Assume that, unknown to the observers, diagnoses can be grouped into two types *M* and *S*, where diagnoses of type *M* is associated with a HRQoL loss of 0.197 (mild) and diagnoses of type *S* one of 0.225 *(*severe). Thus, the measured HRQoL of an individual is (on average) 0.803 for individuals with a mild diagnosis, and 0.775 for individuals with a severe diagnosis. Moreover, assume that severe diagnoses are rare among those individuals with only one diagnosis, say in a 1:9 relationship to the mild ones, while they are ubiquitous among those with two diagnoses. In this world, we would measure the average HRQoL to be $\frac{9 \cdot 0.803\; + \;0.777}{10}\; = \;0.800$ among those with only one diagnosis. How would we interpret a measured average of 0.600 in HRQoL among those with two diagnoses?
Certainly, this estimate fits with an additive model, since the average measured HRQoL loss associated with one diagnosis is 0.200 and 1 – 2 × 0.200 = 0.600. But we also note that 0.775^2^ = 0.600 which fits with a multiplicative model when we take into account that in our example all those who suffer from two diagnoses suffer from two severe ones

In the example above, a multiplicative relationship is disguised as an additive one. By choosing different values and case mixes of mild and severe diagnoses, one can of course construct a model in which the opposite phenomenon is present just as easily.
